# Intra-species variation within *Lactobacillus rhamnosus* correlates to beneficial or harmful outcomes: lessons from the oral cavity

**DOI:** 10.1186/s12864-020-07062-3

**Published:** 2020-09-24

**Authors:** Mangala A. Nadkarni, Nandan P. Deshpande, Marc R. Wilkins, Neil Hunter

**Affiliations:** 1 Institute of Dental Research, Westmead Centre for Oral Health, Westmead Hospital, Sydney, New South Wales Australia; 2 Westmead Institute for Medical Research, The University of Sydney, Sydney, New South Wales Australia; 3 The School of Dentistry, the Faculty of Medicine and Health, The University of Sydney, Sydney, New South Wales Australia; 4 Systems Biology Initiative, School of Biotechnology and Biomolecular Sciences, The University of New South Wales, Sydney, New South Wales Australia; 5 School of Biotechnology and Biomolecular Sciences, The University of New South Wales, Sydney, New South Wales Australia; 6 Ramaciotti Centre for Genomics, The University of New South Wales, Sydney, New South Wales Australia

**Keywords:** *Lactobacillus rhamnosus*, Dental caries, Infection, Genome, Defense, Toxin-antitoxin, Extracellular polysaccharide

## Abstract

**Background:**

The origin of most of the *Lactobacillus rhamnosus* genome sequences lodged in NCBI can be traced to food and faecal isolates followed by blood and tissue sites but with minimal representation from oral and vaginal isolates. However, on the *L. rhamnosus* phylogenetic tree no apparent clade is linked to the origin of isolation or to the relevant clinical source, except for a distinct clade exclusively shared by *L. rhamnosus* isolates from early stages of dental pulp infection (LRHMDP2 and LRHMDP3) and from bronchoalveolar lavage (699_LRHA and 708_LRHA) from a critical care patient. These *L. rhamnosus* strains, LRHMDP2, LRHMDP3, 699_LRHA and 708_LRHA isolated from different continents, display closest genome neighbour gapped identity of 99.95%. The aim of this study was to define a potentially unique complement of genes of clinical relevance shared between these *L. rhamnosus* clinical isolates in comparison to probiotic *L. rhamnosus* strains.

**Results:**

In this analysis we used orthologous protein identification tools such as ProteinOrtho followed by tblastn alignments to identify a novel tyrosine protein phosphatase (wzb)-tyrosine-protein kinase modulator EpsC (wzd)- synteny exopolysaccharide (EPS) cluster. This EPS cluster was specifically conserved in a clade of 5 clinical isolates containing the four *L. rhamnosus* clinical isolates noted above and *Lactobacillus* spp. HMSC077C11, a clinical isolate from a neck abscess. The EPS cluster was shared with only two other strains, *L. rhamnosus* BPL5 and BPL15, which formed a distant clade on the *L. rhamnosus* phylogenetic tree, with a closest genome neighbour gapped identity of 97.51% with *L. rhamnosus* LRHMDP2 and LRHMDP3.

Exclusivity of this EPS cluster (from those identified before) was defined by five EPS genes, which were specifically conserved between the clade of 5 clinical isolates and *L. rhamnosus* BPL5 and BPL15 when compared to the remaining *L. rhamnosus* strains. Comparative genome analysis between the clade of 5 clinical isolates and *L. rhamnosus* BPL5 and BPL15 showed a set of 58 potentially unique genes characteristic of the clade of 5.

**Conclusion:**

The potentially unique functional protein orthologs associated with the clade of 5 clinical isolates may provide understanding of fitness under selective pressure.

## Background

The ubiquitous nature of *L. rhamnosus* in multiple ecological niches including food, gut, oral cavity, vaginal cavity and other tissue sites has inspired many studies of the evolution, niche adaptability and possible safety concerns relating to occasional opportunistic pathogenicity of this species [1–4].

Blood isolates of *L. rhamnosus* [5] and clinical isolates from intensive care unit (ICU) patients [6] showed no apparent clustering on the *L. rhamnosus* phylogenetic tree, while a grouping was reported between *L. rhamnosus* strains from food and faeces [3] within the limited numbers of *L. rhamnosus* genomes accessible through NCBI at the time of that report (2014). Between 2009 and 2019, 172 *L. rhamnosus* genome assemblies became available in NCBI providing an opportunity to review clustering within this species.

**Fig. 1:**
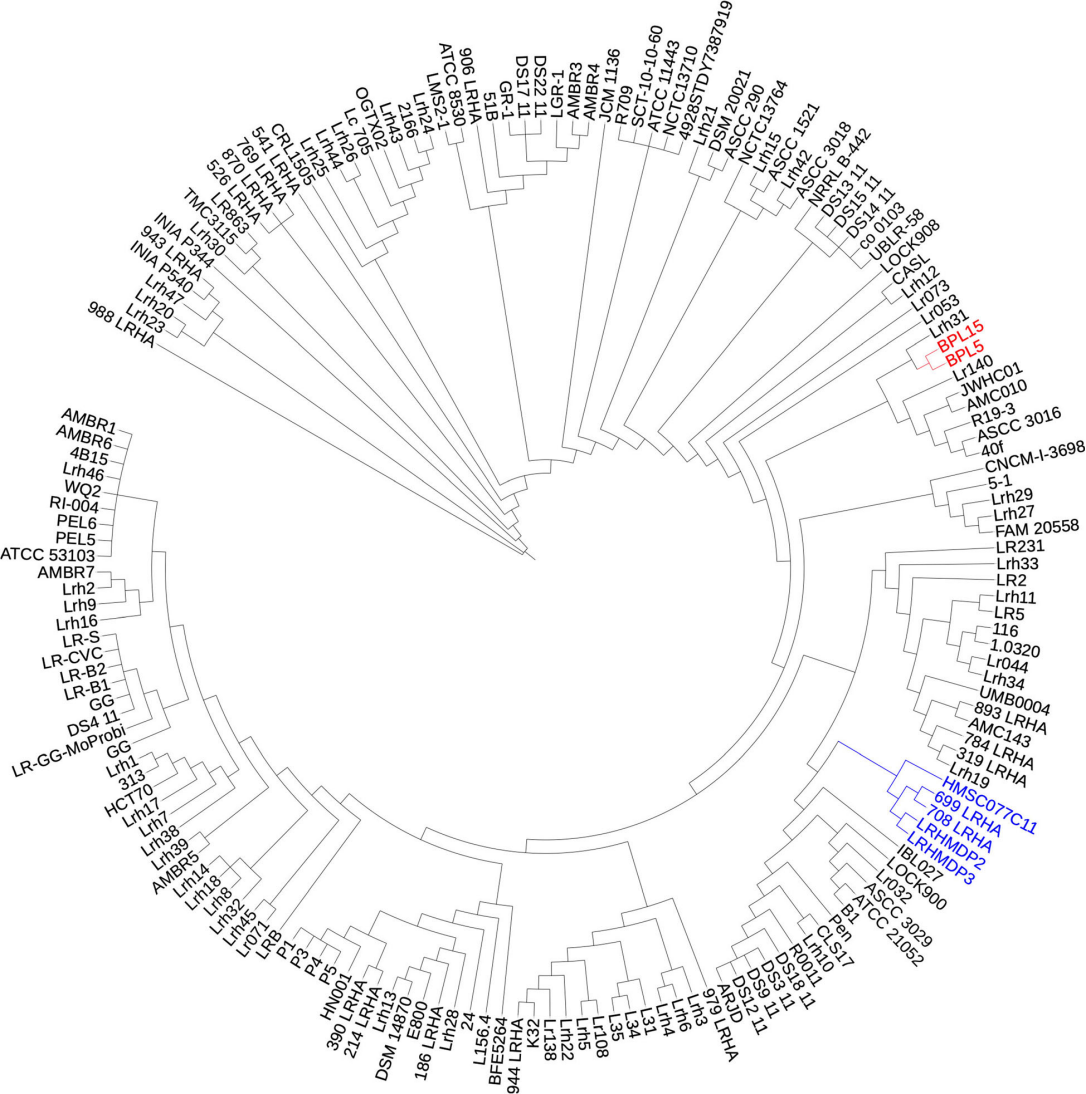
*L. rhamnosus* phylogenetic tree. GToTree was used to generate alignments and a phylogenetic tree based on HMM profiles. For comparative genomics analysis Genbank, fasta and gff format files were downloaded using the NCBI web link https://www.ncbi.nlm.nih.gov/genome/doc/ftpfaq/#downloadservice (Table S1). **Blue:** Genome sequences of *L. rhamnosus* and *Lactobacillus* spp. from the exclusive clinical clade of 5 strains: *L. rhamnosus* LRHMDP2, LRHMDP3, 699_LRHA, 708_LRHA and *Lactobacillus* spp. HMSC077C11. **Red:** *L. rhamnosus* BPL5 and BPL15 form a clade distant from the clinical clade of 5 strains

Analysis indicated that *L. rhamnosus* HN001 from a yoghurt inoculum was seen to group with *L. rhamnosus* E800 isolated from human faeces. *L. rhamnosus* R011 from cheddar cheese grouped with *L. rhamnosus* ATCC 21052 from faeces while *L. rhamnosus* LC705 from milk grouped with *L. rhamnosus* ATCC 8530 isolated from human airways and *L. rhamnosus* LMS2–1 from human gut [3]. Probiotic lactobacilli, *L. rhamnosus* GG and *L. rhamnosus* 53103, isolated from the gut of a healthy individual, grouped with PEL5 and PEL6 sourced from a gut biopsy [3]. It was notable that *L. rhamnosus* LRHMDP2 and *L. rhamnosus* LRHMDP3, isolated from the early stages of infection of dental pulp, formed a distinct cluster [3, 7]. These strains could be categorized as having invasive potential as a significant role for *L. rhamnosus* in the early stages of invasion of vital pulp tissue became apparent in 16S rRNA-based fluorescence in-situ hybridization studies [8]. In the oral cavity, *L. rhamnosus* was implicated in the progression of cavitated carious lesions. Accordingly, the organism was distributed over a broad pH range from an acidic superficial zone of decalcified dentine matrix to a higher pH zone adjacent to vital dental pulp [9]. However, in the initial stages of dental pulp infection the abundance of *L. rhamnosus* expressing copious amounts of exopolysaccharide suggested a pathogenic potential beyond the capacity for production of lactic acid [8, 10–13]. *L. rhamnosus* LRHMDP2 and *L. rhamnosus* LRHMDP3, were the first of the oral isolates to be sequenced [3, 7]. These isolates were segregated from the probiotic strain *L. rhamnosus* GG by 264 and 258 new genes respectively. Differences included a distinctive exopolysaccharide cluster (EPS), transcriptional regulators, an iron ABC transporter and a two component sensor kinase with Ferric iron transporter. Additional differences included the absence of *L. rhamnosus* GG *spa*CBA pilus cluster and of the Clustered Regularly Interspaced Short Palindromic Repeat (CRISPR) - CRISPR associated (cas) system [7]. Genome sequences from 18 *L. rhamnosus* clinical isolates from ICU patients [6] revealed that two *L. rhamnosus* clinical isolates, 699_LRHA and 708_LRHA, isolated 4 days apart from bronchoalveolar lavage of the same patient, were the only clinical isolates to exclusively share the distinct clade with LRHMDP2 and LRHMDP3. Other oral isolates, *L. rhamnosus* LRB from an exfoliated deciduous tooth [14] and *L. rhamnosus* 24, 308 and 313 from infant and adult saliva (closest genome neighbour gapped identity of 97.4% with *L. rhamnosus* LRHMDP2 and LRHMDP3) as well as other clinical isolates from a variety of tissue sources, showed random distribution on the *L. rhamnosus* phylogenetic tree as displayed in Fig. 1.

Apart from the significant association of *L. rhamnosus* with progression of dental caries, and as an occasional opportunistic pathogen in infective endocarditis in patients with cardiac risk factors [15, 16], a major focus on *L. rhamnosus* has been on probiotic properties. One of the criteria to be generally recognized as safe (GRAS) and having beneficial effects on the host, is the absence of an inflammatory response [17]. Accordingly, the focus on probiotic action of lactobacilli in humans is on released products and on surface components, particularly exopolysaccharides (EPS) of capsular or cell wall origin [18]. Probiotic *L. rhamnosus* LOCK 900 has been shown to express both low and high molecular weight exopolysaccharides with distinct structures and biological properties [19]. Similarly, the genome of *L. rhamnosus* DSM 14870 from the prophylactic EcoVag capsule was found to encode two putative EPS clusters. EPS cluster 1 exhibited identity to *L. rhamnosus* Lc705 EPS cluster while the majority of the ORFs of the EPS cluster 2 showed identity to *L. rhamnosus* HN001 EPS cluster [20]. Similarly, EPS clusters of other *L. rhamnosus* probiotic and clinical isolates have also shown considerable divergence [7, 18, 21, 22]. Blood isolates from cluster A (*n* = 8) shared the EPS cluster of *L. rhamnosus* GG whereas other blood isolates from cluster B (*n* = 7) were found to possess a different EPS cluster [5]. *L. rhamnosus* strains (*n* = 40) from diverse environments such as fermented dairy products, beer, animal and human faeces, blood and vagina, group into six EPS gene clusters with one of the EPS clusters formed by multiple predicted mannosyl-glycosyltransferases considered to synthesise mannosyl-EPS [1]. Beyond the focus on the EPS cluster, niche adaptation was also attributed to an accessory genome comprising pilus gene clusters, CRISPR - cas system genes, carbohydrate transport and metabolism genes, bacteriocin production and mobile genetic elements [1].

In the present study, using *in-silico* analysis, we highlight a potentially unique complement of genes of clinical relevance in the clade of 5 clinical isolates (*L. rhamnosus* LRHMDP2, LRHMDP3, 699_LRHA, 708_LRHA and *Lactobacillus* spp. HMSC077C11) in comparison to the probiotic strains *L. rhamnosus* BPL5 and BPL15. Except for the near identical EPS cluster, with exclusivity defined by five EPS orthologs, no other genes were specifically conserved between the clade of 5 clinical isolates and the probiotic strains, *L. rhamnosus* BPL5 and BPL15 as compared to other *L. rhamnosus* strains.

A set of 58 genes with known biological functions were found to be specifically conserved across the clade of 5 clinical isolates when compared to the *L. rhamnosus* BPL5 and BPL15 strains. Of the 58 genes, 14 genes with important biological functions were found to be orthologous across the clade of 5 clinical isolates with minimal presence (1–7 strains) across the remaining *L. rhamnosus* strains.

## Results

### A unique clade of 5 clinical isolates of *L. rhamnosus*

With no apparent clades reflecting the origin of isolation within a phylogeny tree comprising 172 sequenced isolates (Fig. 1, Table S1), it was considered important to decipher the genomic features of *L. rhamnosus* isolates that represented a unique clade on the *L. rhamnosus* phylogenetic tree.

*L. rhamnosus* isolates from the early stages of dental pulp infection (LRHMDP2 and LRHMDP3) which showed closest genome neighbour gapped identity of 99.95% with *L. rhamnosus* isolates (699_LRHA and 708_LRHA) from bronchoalveolar lavage, shared a distinct clade on the *L. rhamnosus* phylogeny tree (Fig. 1). *L. rhamnosus* 699_LRHA and 708_LRHA were isolated 4 days apart from the same patient from a polymicrobial infection that included *Escherichia coli* and *Serratia marcescens* [6]. Inclusion of *Lactobacillus* spp. HMSC077C11 in the present study was incidental based on an identical protein search on NCBI for the *L. rhamnosus* LRHMDP2 and LRHMDP3 EPS cluster protein orthologs (Table 1). The *Lactobacillus* spp. HMSC077C11 genome sequence was lodged in NCBI as an unnamed isolate not characterized using traditional culture identification methods and being clearly distinct from currently identified species (https://www.ncbi.nlm.nih.gov/genome/?term=HMSC077C11). However, a recent genome- based species taxonomy study re-classified *Lactobacillus* spp. HMSC077C11 as *Lactobacillus rhamnosus* in the Genome Taxonomy Database (GTDB) [23]. Our analysis also showed that *Lactobacillus* spp. HMSC077C11 occupied the same clade as *L. rhamnosus* LRHMDP2, LRHMDP3, 699_LRHA and 708_LRHA on the *L. rhamnosus* phylogenetic tree (Fig. 1).

**Table 1:** Representation of wzb-wzd synteny EPS cluster ^*a*^ of *L. rhamnosus* LRHMDP2, LRHMDP3 in *L. rhamnosus* 699_LRHA, 708_LRHA, *Lactobacillus* spp. (*L. rhamnosus*) HMSC077C11 and *L. rhamnosus* BPL5, BPL15

ORF	Gene Description	HMSC077C11	699_LRHA	708_LRHA	LRHMDP2	LRHMDP3	BPL15	BPL5	Number of ***L. rhamnosus*** strains with the protein ortholog
wzb	tyrosine protein phosphatase	WP_015765006.1					WP_061713774.1		166
wzr	Cell envelope-associated transcriptional attenuator LytR-CpsA-Psr, subfamily F2	WP_005715254.1					WP_061713383.1		166
welE	sugar transferase	WP_070586506.1	WP_049168896.1		WP_005715255.1		WP_061713382.1		152
hypothetical	hypothetical protein	WP_005715256.1					WP_061713381.1		66
**lipopolysaccharide biosynthesis protein** ^***b***^	**lipopolysaccharide biosynthesis protein**	**WP_005715258.1**					**P** ^***c***^	**WP_061713380.1**	**7**
**wchA** ^***b***^	**glycosyltransferase family 2 protein**	**WP_005715259.1**							**7**
**Polysaccharide transferase protein** ^***b***^	**polysaccharide pyruvyl transferase family protein**	**WP_005715260.1**		**P** ^***c***^	**WP_005715260.1**		**WP_061713379.1**		**7**
welL	glycosyltransferase family 2 protein	WP_005715262.1					WP_061713378.1		48
**Hypothetical** ^***b***^	**hypothetical protein**	**P** ^***c***^	**P** ^***c***^	**P** ^***c***^	**WP_005715263.1**	**P** ^***c***^	**P** ^***c***^	**P** ^***c***^	**7**
**Polymerase** ^***b***^	**oligosaccharide repeat unit polymerase**	**WP_005717894.1**		**P** ^***c***^	**WP_005715264.1**	**WP_005717894.1**	**P** ^***c***^	**P** ^***c***^	**7**
hypothetical protein	hypothetical protein	WP_050562870.1	WP_049175362.1	WP_049168568.1	WP_050562870.1		WP_081014187.1	WP_050562870.1	34
WelJ/WciB	DUF4422 domain-containing protein	WP_005715265.1					WP_061713377.1		84
glf	UDP-galactopyranose mutase	WP_005715266.1							89
wze	polysaccharide biosynthesis tyrosine autokinase (CpsD/CapB family tyrosine-protein kinase)	WP_005715267.1					WP_061713376.1		162
wzd	Tyrosine-protein kinase modulator EpsC	WP_005715268.1					WP_061713375.1		165

^a^: wzb-wzd synteny EPS cluster identified using the protein othology tool ProteinOrtho,

^b^: Genes specifically conserved in *L. rhamnosus* LRHMDP2, LRHMDP3, *L. rhamnosus* 699_LRHA, 708_LRHA, *Lactobacillus* spp. HMSC077C11 and *L. rhamnosus* BPL5, BPL15 (**Bold**)

^c^: The genes marked as “P” were also found to be present but had to be identified using tblastn alignments against genomes and hence “WP_” identifiers from NCBI are not available (**Bold**)

### A novel EPS cluster shared across the clade of 5 *L. rhamnosus* clinical isolates and the *L. rhamnosus* strains BPL5 and BPL15

A targeted search on NCBI for Identical Protein groups of *L. rhamnosus* LRHMDP2 and LRHMDP3 wzb-wzd synteny EPS cluster led to the finding of a conserved EPS cluster in the clade of 5 clinical isolates. This finding offered a critical basis to search for conservation of the EPS cluster orthologs across other *L. rhamnosus* strains. Of the 172 *L. rhamnosus* isolates whose sequences are included in this study, only two others, *L. rhamnosus* strains, BPL5 (CECT 8800) a vaginal probiotic [24] and *L. rhamnosus* BPL15 (CECT 8361) [25], which form a distant clade, shared a near identical EPS cluster with the clade of 5 clinical isolates. Both *L. rhamnosus* strains, BPL5 and BPL15, showed a closest genome neighbour gapped identity of 97.51% with *L. rhamnosus* LRHMDP2 and LRHMDP3 and 97.49% with *L. rhamnosus* 699_LRHA and 708_LRHA. Therefore, these two strains were also included in the analysis.

**Fig. 2:**
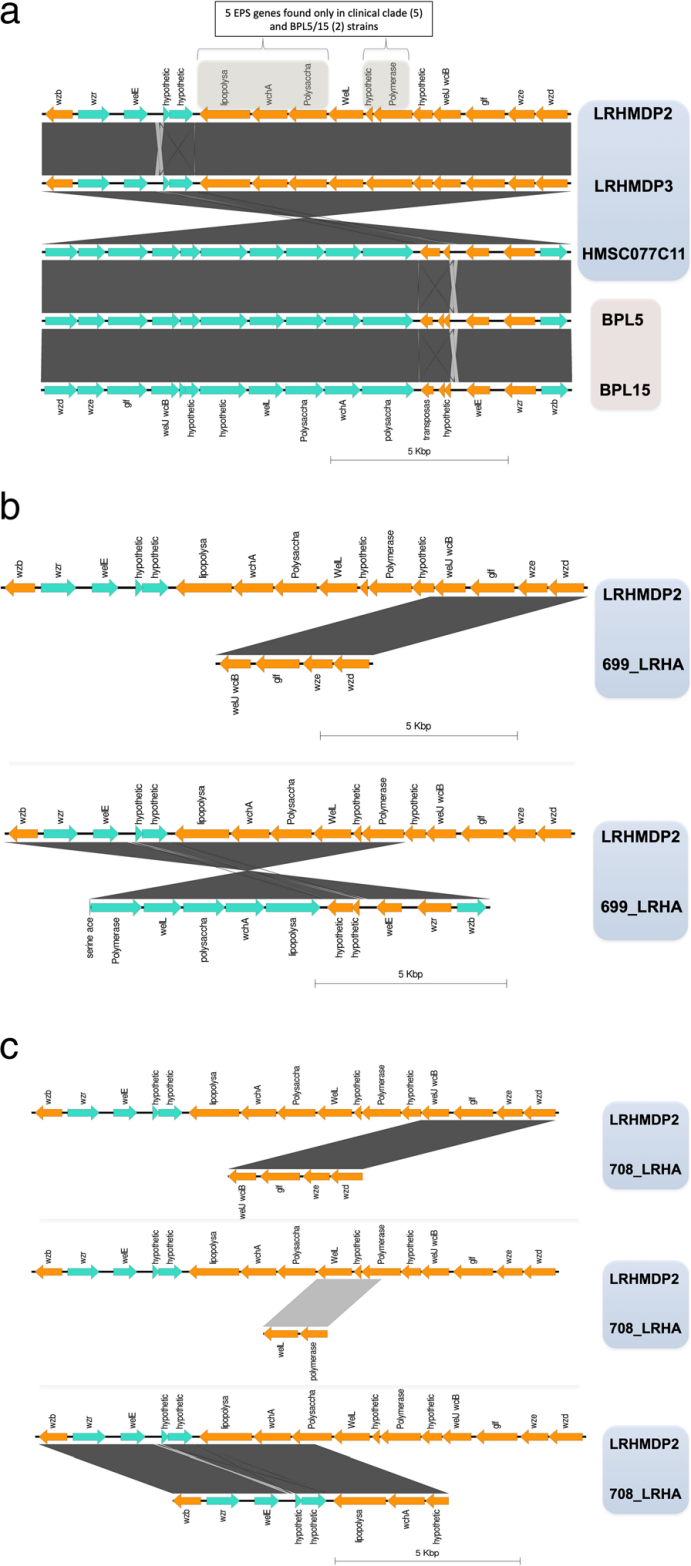
Genomic organisation of wzb-wzd synteny EPS cluster of *L. rhamnosus* LRHMDP2, LRHMDP3, *L. rhamnosus* 699_LRHA, 708_LRHA, *Lactobacillus* spp. HMSC077C11 and *L. rhamnosus* BPL5/BPL15. Proteinortho, a standalone tool, was used to determine conservation of orthologous genes across selected *L. rhamnosus* isolates for a specific EPS gene cluster. Genomic organization of EPS cluster genes was ascertained using EasyFig, a python application for creating linear comparison figures of multiple genomic loci, within the ‘wzb-wzd’ synteny EPS cluster locus. *L. rhamnosus* LRHMDP2, LRHMDP3, BPL5, BPL15 and *Lactobacillus* spp. HMSC077C11 on continuous contig (**a**), *L. rhamnosus* LRHMDP2, LRHMDP3 and 699_LRHA split on to 2 contigs (**b**). *L. rhamnosus* LRHMDP2, LRHMDP3 and 708_LRHA (split on to 3 contigs) (**c**)

Five genes from the wzb-wzd bound EPS cluster, a gene encoding a homologue of lipopolysaccharide biosynthesis protein, a glycosyltransferase family 2 protein (wchA), a polysaccharide pyruvyl transferase family protein, a hypothetical protein and an oligosaccharide repeat unit polymerase, were syntenically conserved specifically in these seven strains (the clade of 5 clinical isolates and *L. rhamnosus* BPL5 and BPL15) when compared to all other *L. rhamnosus* strains (Table 1). The five genes are thus exclusive to this EPS cluster. The remainder of the elements of the wzb-wzd EPS cluster were found to be conserved across many other *L. rhamnosus* strains. The tool EasyFig was used to ascertain the genomic organization of EPS cluster genes displaying similarities and differences in the orientation within the ‘wzb-wzd’ synteny EPS cluster across the clade of 5 clinical isolates and BPL5 and BPL15 (Fig. 2a, b, c. The green and orange colours specify gene orientations). Near identical genomic organisation and orientation of EPS cluster genes between the two dental pulp isolates, *L. rhamnosus* LRHMDP2 and LRHMDP3, was apparent [7] except for an inversion in the intergenic region adjacent to the welE gene and the presence of an additional hypothetical protein in LRHMDP2 as compared to LRHMDP3 (Fig. 2a). Similarly, genomic organisation and orientation of EPS cluster genes between *L. rhamnosus* BPL5 and BPL15 also remained near identical but for an inversion in the intergenic region (adjacent to the welE gene) as was apparent in LRHMDP2 and LRHMDP3 (Fig. 2a). *Lactobacillus* spp. HMSC077C11 EPS cluster genes showed consensus with *L. rhamnosus* BPL5 and BPL15 for genomic organisation and orientation except for a missing gene adjacent to the inversion region (Fig. 2a). However, the EPS cluster genes of *L. rhamnosus* BPL5, BPL15 and *Lactobacillus* spp. HMSC077C11 showed inverted orientation as compared to *L. rhamnosus* LRHMDP2 and LRHMDP3 (Fig. 2a). When the genomic context of the relevant assemblies was examined, the EPS cluster genes of *L. rhamnosus* LRHMDP2, LRHMDP3 and *Lactobacillus* spp. HMSC077C11 could be detected on the same contig whereas EPS cluster genes of *L. rhamnosus* 699_LRHA were found to be split between two different contigs (Fig. 2b) and 708_LRHA EPS cluster genes were found split between three different contigs (Fig. 2c). In *L. rhamnosus* 699_LRHA orientation of some of the EPS cluster genes was inverted compared to *L. rhamnosus* LRHMDP2 and LRHMDP3. *L. rhamnosus* 708_LRHA maintained orientation of the EPS cluster genes in the same order as for *L. rhamnosus* LRHMDP2 and LRHMDP3.

### Genomic features which distinguish the clinical clade of 5 *L. rhamnosus* isolates

Having established that the EPS cluster was common between the clinical clade of *L. rhamnosus* and strains BPL5 and BPL15, we sought to determine whether there were other features that were also common between these strains. Consistent with the substantial genomic distance between the clade of 5 clinical isolates and *L. rhamnosus* BPL5 and BPL15, no other gene-sets were specifically conserved between *L. rhamnosus* BPL5, BPL15 and the clade of 5, except for the near identical EPS cluster, with exclusivity defined by 5 EPS orthologs.

Using Proteinortho (protein sequences) and tblastn (genome sequence), 107 proteins were found to be orthologous across *L. rhamnosus* LRHMDP2, LRHMDP3, 699_LRHA, 708_LRHA and *Lactobacillus* spp. HMSC077C11 and to be absent in *L. rhamnosus* BPL5 and BPL15 (Table S2). Protein function could be attributed to 58 entities while 49 were categorised as hypothetical proteins (Table S2). These proteins separating the clade of 5 clinical isolates and *L. rhamnosus* BPL5 and BPL15 included an ABC-2 transporter permease, alpha/beta hydrolase, cytosine permease, DUF917 domain-containing protein, hydantoinase/oxoprolinase family protein, N-acetyltransferase and SDR family NAD(P)-dependent oxidoreductase, all of which were also present in the majority of the other *L. rhamnosus* strains (Table 2). In addition, five genes of the six gene cassette of the *Bacillus cereus* phage defense system, BREX (bacteriophage exclusion), shown to confer resistance to integration of lysogenic (temperate) phages as well as replication of lytic phages [26, 27] could also be identified as a distinct feature of clade of 5 as compared to *L. rhamnosus* BPL5 and BPL15. BREX system orthologs of clade of 5 clinical isolates could be detected in other *L. rhamnosus* strains mainly from human origin and including vaginal isolates GR-1, 51B and DSM 14870 but not Lrh31 (Table S3). The clade of 5 BREX system orthologs could also be identified in most of the blood isolates (except for Lrh15 and Lrh47) including LRB1 and LRB-2 infant blood isolates and in the central venous catheter isolate from the *L. rhamnosus* GG clade. Similarly, presence of BREX system orthologs in clinical isolates from ICU patients could be grouped with other blood isolates. However, clade of 5 BREX system orthologs were not detected in the clinical isolates 526_LRHA, 541_LRHA, 769_LRHA, 879_LRHA, 943_LRHA and 988_LRHA from ICU patients, on a clade shared with blood isolate Lrh47. Absence of BREX system orthologs was also notable in isolates from infant saliva (*L. rhamnosus* 24) and from infant stools (*L. rhamnosus* L31, L34 and L35) (Table S3).

**Table 2:** Distinct functional protein orthologs associated with clade of 5 *L. rhamnosus* clinical isolates in comparison to *L. rhamnosus* BPL5, *L. rhamnosus* BPL15 and the rest of the *L. rhmanouss* genomes

Description	Protein ID	# ***L. rhamnosus*** Species
4-hydroxy-2-oxoglutarate aldolase / 2-dehydro-3-deoxyphosphogluconate aldolase	WP_005717709.1	77
ABC-2 transporter permease	WP_005715415.1	164
AbrB/MazE/SpoVT family DNA-binding domain-containing protein	WP_005715999.1	54
alpha-L-fucosidase	WP_005717725.1	81
alpha/beta hydrolase	WP_005715325.1	152
AraC family transcriptional regulator	WP_005714953.1	60
bacteriocin	WP_076638842.1	59
beta-galactosidase subunit alpha	WP_005714949.1	68
BREX system Lon protease-like protein BrxL	WP_005686196.1	91
BREX system P-loop protein BrxC	WP_005715305.1	89
BREX-1 system phosphatase PglZ type A	WP_005686195.1	87
carbohydrate PTS IIA component	WP_005715915.1	76
cytosine permease	WP_005715299.1	146
DNA helicase	WP_049168901.1	44
DUF1788 domain-containing protein (brxB)	WP_005684780.1	82
DUF1819 domain-containing protein (brxA)	WP_005684779.1	82
DUF2568 domain-containing protein	WP_005716418.1	53
DUF262 domain-containing protein sp. HMSC077C11	WP_070586510.1	91
DUF2620 domain-containing protein	WP_005714931.1	37
DUF2992 domain-containing protein	WP_005685750.1	97
DUF917 domain-containing protein	WP_005715298.1	151
galactonate dehydratase	WP_005717713.1	77
GntR family transcriptional regulator	WP_005717726.1	78
helix-turn-helix domain-containing protein (Rgg/GadR/MutR family transcriptional regulator)	WP_032954331.1	45
HXXEE domain-containing protein	WP_005716405.1	80
hydantoinase/oxoprolinase family protein	WP_005715297.1	144
L-fucose isomerase	WP_005717692.1	74
membrane protein	WP_005714932.1	38
MerR family transcriptional regulator sp. HMSC077C11	WP_070586464.1	53
metal-independent alpha-mannosidase	WP_005717706.1	73
N-acetyltransferase	WP_005715414.1	164
nucleoside-diphosphate sugar epimerase	WP_005717601.1	32
phosphotriesterase-related protein sp. HMSC077C11	WP_070586459.1	41
PRD domain-containing protein	WP_032954335.1	21
PRD domain-containing protein	WP_005714930.1	37
pyridoxamine 5-phosphate oxidase family protein	WP_015764910.1	67
SDR family NAD(P)-dependent oxidoreductase	WP_005684771.1	152
SEC10/PgrA surface exclusion domain-containing protein sp. HMSC077C11	WP_070586541.1	35
thymidylate synthase	WP_032954600.1	96
transaldolase	WP_005714910.1	37
transketolase	WP_005714895.1	43
type 1 glutamine amidotransferase domain-containing protein	WP_005717600.1	31
type III restriction protein res subunit (DEAD/DEAH box helicase)	WP_005716818.1	69
YhfX family PLP-dependent enzyme	WP_005714936.1	38

In *L. rhamnosus* isolates of human origin a link was evident between the presence or absence of clade of 5 BREX system orthologs and alpha-L-fucosidase, L-fucose isomerase and metal-independent alpha-mannosidase (Table 2, Table S3). The *L. rhamnosus* clinical isolates and other isolates of human origin with detectable BREX system orthologs also showed presence of alpha-L-fucosidase, L-fucose isomerase and metal-independent alpha-mannosidase. Exceptions included a clade shared between Lrh30, Lrh23, Lrh20 (blood isolates), 906_LRHA (clinical isolate) and GR-1 and 51B (vaginal isolates) which showed absence of alpha-L-fucosidase, L-fucose isomerase and metal-independent alpha-mannosidase despite detectable orthologs of clade of 5 BREX system orthologs. In contrast, another vaginal isolate *L. rhamnosus* DSM14870 showed presence of clade of 5 BREX system orthologs and alpha-L-fucosidase, L-fucose isomerase and metal-independent alpha-mannosidase. These genes were not detected in vaginal isolate Lrh31. This was similar to the profile for *L. rhamnosus* BPL5 and BPL15 and for clinical isolates from ICU patients (526_LRHA, 541_LRHA, 769_LRHA, 879_LRHA, 943_LRHA and 988_LRHA) and blood isolate Lrh 47. Alpha-L-fucosidase, L-fucose isomerase, metal-independent alpha-mannosidase and BREX system orthologs could be detected in other oral and salivary isolates, *L. rhamnosus* LRB and 313. In *L. rhamnosus* 24, an isolate from infant saliva, alpha-L-fucosidase and L-fucose isomerase were detected but BREX system orthologs were absent (Table 2, Table S3).

An identical pattern of distribution across the *L. rhamnosus* strains of human origin was also evident for HXXEE domain-containing protein and thymidylate synthase with alpha-L-fucosidase, L-fucose isomerase and metal-independent alpha-mannosidase and presence or absence of clade of 5 BREX system orthologs (Table 2, Table S3).

In contrast to  the clade of 5, AbrB/MazE/SpoVT family DNA-binding domain-containing protein from the toxin-antitoxin (TA) defence system was absent in the *L. rhamnosus* isolates from *L. rhamnosus* GG clade (including blood isolates, LR-CVC and oral isolate LRB), Lrh30, Lrh23 and Lrh20 (blood isolates), 906_LRHA (clinical isolate) and GR-1and 51B (vaginal isolate) and salivary isolate 313, but was present in infant salivary isolate *L. rhamnosus* 24. AbrB/MazE/SpoVT family DNA-binding domain-containing protein could not be detected in the clinical isolates from ICU patients, (526_LRHA, 541_LRHA, 769_LRHA, 879_LRHA, 943_LRHA and 988_LRHA) on a shared clade with blood isolate Lrh47. However, the blood isolates, Lrh13, Lrh28, 186_LRHA, 214_LRHA, 390_LRHA on a clade shared with DSM 14870 (vaginal probiotic) and Lrh11, 319_LRHA, 784_LRHA and 893_LRHA showed presence of AbrB/MazE/SpoVT family DNA-binding domain-containing protein from the clade of 5 clinical isolates.

A set of 14 potentially unique functional orthologs displayed orthologous conservation in the clade of 5 clinical isolates and were conserved across a limited number (between 1 and 7) of other *L rhamnosus* isolates. Notable being transcriptional regulators, RNA polymerase sigma-54 factor, XRE family transcriptional regulator, PRD domain-containing protein WP_005714928.1(earlier classified as NtrC transcriptional regulator [7]); also, Iron ABC transporter substrate-binding protein, iron ABC transporter permease, and two component sensor kinase with Ferric iron transporter, ImmA/IrrE family metallo-endopeptidase, IS91 family transposase, isochorismatase and ATP-binding protein (Table 3).

**Table 3:** Exclusive set of unique functional protein orthologs in clade of 5 *L. rhamnosus* clinical isolates in comparison to *L. rhamnosus* BPL5, *L. rhamnosus* BPL15 and the rest of the *L. rhamnosus* genomes

Gene description	Protein IDs	# ***L. rhamnosus*** Species
ATP-binding protein	WP_005717598.1	9
DUF4260 domain-containing protein	WP_080600050.1	8
helix-turn-helix domain-containing protein (Rgg/GadR/MutR family transcriptional regulator)	WP_005717505.1	12
ImmA/IrrE family metallo-endopeptidase	WP_005715904.1	9
iron ABC transporter permease	WP_005716278.1	6
iron ABC transporter substrate-binding protein	WP_005716273.1	6
IS91 family transposase	WP_005717838.1	8
isochorismatase	WP_005715911.1	6
phage portal protein	WP_005716841.1	10
PRD domain-containing protein (NtrC transcription regulator)	WP_005714928.1	7
RNA polymerase sigma-54 factor	WP_005714927.1	8
site-specific integrase	WP_080600030.1	5
Two-component sensor kinase associated with ferric iron transporter	WP_005716270.1	6
XRE family transcriptional regulator (helix-turn-helix transcriptional regulator)	WP_005715906.1	9

XRE family transcriptional regulator and ImmA/IrrE family metallo-endopeptidase were exclusively shared with L34, L35, L31 (infant stool isolates) and Lrh22 (blood isolate), RNA polymerase sigma-54 factor with L34, L35 and L31 and PRD domain-containing protein (NtrC transcription regulator) only with L34 and L35.

Iron ABC transporter substrate-binding protein, iron ABC transporter permease, two component sensor kinase with Ferric iron transporter and isochorismatase were highly conserved and exclusively shared orthologs between clade of 5 clinical isolates and L31 from infant faeces.

In addition to the clade of 5 clinical isolates, IS91 family transposase was detected only in JWHC01 (*L. rhamnosus* strain 308 from saliva of a healthy female), AMC010 from stools of a healthy infant and E800 (from faeces). IS91 family transposase (identified earlier as a putative transposase) was found to be surrounded by a site-specific integrase, a phage portal protein and eight distinctive clade -specific hypothetical proteins (WP_005716827 - WP_005716850) and WP_032954616 (Table S2).

## Discussion

We used the availability of a large number of *L. rhamnosus* genome sequences present in NCBI to uncover a unique, clinically relevant clade comprising *L. rhamnosus* LRHMDP2 and LRHMDP3 from dental pulp infection together with *L. rhamnosus* isolates 699_LRHA and 708_LRHA from bronchoalveolar lavage of a critical care patient [6]. The four *L. rhamnosus* clinical isolates shared closest genome neighbour gapped identity of 99.95% and also shared the unique tyrosine protein phosphatase (wzb)-tyrosine-protein kinase modulator EpsC (wzd) synteny exopolysaccharide (EPS) cluster of *L. rhamnosus* LRHMDP2 and LRHMDP3 [7]. In addition, *Lactobacillus* spp. HMSC077C11, a clinical isolate from a neck abscess, was re-classified as *Lactobacillus rhamnosus* in the Genome Taxonomy Database (GTDB) [23]. This isolate occupied the same clade as *L. rhamnosus* LRHMDP2, LRHMDP3, 699_LRHA and 708_LRHA on the *L. rhamnosus* phylogenetic tree and shared the exclusive EPS cluster of *L. rhamnosus* LRHMDP2 and LRHMDP3.

A primary environmental niche for the clade of 5 has not been identified. Findings do not exclude an oral cavity source, particularly for isolates recovered from bronchoalveolar lavage but also from a neck abscess. A distinguishing genomic feature of this clade is an exopolysaccharide cluster. While a role for the exopolysaccharides of this clade in pathogenicity remains to be investigated, our preliminary studies, using partially purified polysaccharide extracted from isolate LRHMDP2, revealed perturbation of adaptive neurogenesis (data not shown). In context, there is a profound neural response in dental pulp tissue in response to microbial invasion of dentine [28]. Disruption of this adaptive response compromises defense allowing bacteria to invade dental pulp tissue. Modulation of neural function has been reported for bacterial polysaccharides [29] and this could represent an important aspect of the beneficial action of probiotic lactobacilli, particularly as there is limited evidence for effective colonisation of the adult human gut by these organisms [29].

Capsular polysaccharides and cell wall exopolysaccharides are significant for bacterial pathogenesis apart from potential contribution to the probiotic action of *L. rhamnosus* gut and vaginal isolates [18–22]. It is possible that differences in the relative orientation of EPS cluster genes within the clade of 5 could alter amounts and composition of exopolysaccharide in response to nutrient availability [30] and immune surveillance. The nature of the immune response and the property of adherence and biofilm formation has been shown to be influenced by minor variation in the structure of polysaccharide [31].

A finding in this study was the presence, in *L. rhamnosus* probiotic strains BPL5 and BPL15, of an inversion of the wzb-wzd EPS cluster present in *L. rhamnosus* LRHMDP2 and LRHMDP3. The inverted orientation of the EPS cluster and small differences in the functional orthologs could result in an altered exopolysaccharide in *L. rhamnosus* BPL5 and BPL15. These properties together with the added benefit of enhanced acidogenic capacity could be resultant contributors to the probiotic properties of *L. rhamnosus* BPL5 [32]. Divergence in the organisation of the EPS gene cluster and in composition of the exopolysaccharide among *L. rhamnosus* strains could hold significance for probiotic or pathological action [1, 5, 18–22].

The differences between *L. rhamnosus* BPL5 and BPL15 and the clade of 5, that share near identical EPS clusters, were further investigated. In addition to the EPS cluster, niche adaptation has also been attributed to the accessory genome comprising pilus gene clusters, CRISPR - cas system genes, carbohydrate transport and metabolism genes, bacteriocin production and mobile genetic elements [1]. Comparative genome analysis between the clade of 5 and *L. rhamnosus* BPL5 and BPL15 enabled identification of an exclusive set of 58 functionally identified protein orthologs. These included clade of 5 specific BREX system orthologs, alpha-L-fucosidase, L-fucose isomerase, metal-independent alpha-mannosidase, HXXEE domain-containing protein, thymidylate synthase and AbrB/MazE/SpoVT family DNA-binding domain-containing protein.

The *Bacillus cereus* BREX system contributed one of the distinctive genomic features shared between the clade of 5 in comparison to *L. rhamnosus* BPL5 and BPL15*.* The BREX defense system in *L. rhamnosus* LRHMDP2 and LRHMDP3 consisted of a full length BREX-1 system adenine specific DNA-methytransferase PglX (1198aa) and a partial form of PglX (743aa) separated by a site-specific integrase. This system potentially confers resistance against a broad range of phages. The genomic arrangement is analogous to the BREX system type 1 of *L. rhamnosus* GG [27]. Five of the six-gene-cassette BREX defense system genes from the clade of 5 clinical isolates could be detected in many of the *L. rhamnosus* strains of human origin. These included multiple blood isolates, ICU isolates, probiotic strains and some of the oral and vaginal isolates. In the clade of 5, BREX system P-loop protein BrxC was identified as part of the exclusive set of functionally identified proteins. Although the P-loop-containing gene was conserved, shared homology in various BrxC from six BREX subtypes is low [27]. These findings imply importance for the BREX defense system across a spectrum of *L. rhamnosus* isolates. Another defense strategy, the CRISPR-cas system for control of phages, was shown to be absent in *L. rhamnosus* LRHMDP2 and LRHMDP3 [7]. Absence of the entire CRISPR-cas system was also apparent in *L. rhamnosus* Lc705, ATCC 8530, ATCC 7469 and 8 of 40 diverse *L. rhamnosus* isolates [1, 33]. However, both of the phage resistance systems, CRISPR-cas and BREX, are present in *L. rhamnosus* GG [27, 33]. The trade-off between presence and absence of CRISPR-cas for virulence is apparent in the human pathogen *Streptococcus pneumoniae* [34]. On the other hand, heterogeneity in PglX of the BREX system has been shown to control phase variation in bacterial defense systems to overcome toxic effects of certain genes [27].

Another distinctive feature of the clade of 5 clinical isolates is the antitoxin of the toxin-antitoxin (TA) defense system (AbrB/MazE/SpotVT family type DNA binding protein). This protein was absent from most other *L. rhamnosus* isolates. MazF and YaFQ TA systems have also been reported for *L. rhamnosus* isolates from clade 1 comprising isolates from blood, faeces and other clinical samples [1]. In *E. coli*, chromosomally located MazE antitoxin, a DNA binding protein, has been shown to wrap around the MazF toxin, an endonuclease [35]. Six additional TA systems were identified in *L. rhamnosus* intestinal, faecal and salivary isolates [36]. *L. rhamnosus* LRHMDP2 and LRHMDP3 were found to possesses five of the six TA systems whereas *L. rhamnosus* GG contained three of the six TA systems [36]**.** By reacting to multiple stress factors that a pathogen encounters in the host, TA systems are considered to modulate the host-pathogen interface [37]. Therefore, AbrB/MazE/SpotVT family type DNA binding proteins may signify a causal role for the clade of 5 in clinical pathology.

Another ortholog specific to the clade of 5 was alpha L-fucosidase, found to participate in the degradation of various fucosyl-glycoconjugates on epithelial cell surfaces and in blood group antigens, intestinal mucin and human milk [38]. Release of α-linked fucose residues could provide a source of carbon for the clade of 5 in clinical conditions. The oral isolates *L. rhamnosus* LRHMDP2 and LRHMDP3 were identified as having an L-fucose fermenting phenotype with *fuc* clusters like those of *L. rhamnosus* GG and HN001 as opposed to the absence of fucose fermenting ability in the dairy isolates [39]. Alpha L-fucosidase along with metal-independent alpha-mannosidase could empower the clinical isolates to de-cap and harvest human glycans as evident in *Streptococcus pneumoniae* [40]. Also, within the clade of 5, a GntR family transcriptional regulator was found adjacent to the gene encoding Alpha L-fucosidase. GntR family transcription regulators are termed as sugar transport system regulators in *Streptococcus mutans* [41]. These regulators were deduced to regulate multiple sugar transport genes, EPS production and biofilm formation [41]. Similarly, involvement of GntR type transcription factors in the regulation of the GalN/GalNAc utilization pathway is required for the virulence of *Streptococcus suis* serotype 2 [42]. Therefore, the clade of 5 - specific GntR family transcriptional regulators may play an important role in sugar transport systems, EPS and biofilm formation, in specified niches.

A set of 14 potentially unique functional orthologs could be closely associated with the clade of 5 clinical isolates. Notable inclusions are RNA polymerase sigma-54 factor, XRE family transcriptional regulator, PRD domain-containing protein WP_005714928.1 (earlier classified as a NtrC transcription regulator [7]), iron ABC transporter substrate-binding protein, iron ABC transporter permease, a two component sensor kinase with ferric iron transporter, ImmA/IrrE family metallo-endopeptidase, IS91 family transposase, isochorismatase and an ATP-binding protein. Comparative genomic analysis between *L. rhamnosus* LRHMDP2, LRHMDP3 and *L. rhamnosus* GG also identified RNA polymerase sigma-54 factor (RpoN), transcriptional regulators, NtrC and MutR, an iron ABC transporter permease, iron ABC transporter substrate and a two component sensor kinase with ferric iron transporter, as features of significance [7] within the clade of 5.

The set of 14 potentially unique functional orthologs closely associated with the clade of 5 could not be found in most of the blood isolates (except for Lrh22) or in most of the clinical isolates (except for 944_LRHA) but were found in *L. rhamnosus* L34, L35 and L31 (isolates from infant faeces). XRE family transcriptional regulator and ImmA/IrrE family metallo-endopeptidase were exclusively shared with L34, L35, L31 and Lrh22 (blood isolate) and RNA polymerase sigma-54 factor with L34, L35 and L31 and PRD domain-containing protein (NtrC transcriptional regulator) only with L34 and L35. *L. rhamnosus* L34, L35 (from a 40 day old infant) and L31 (from a 39 day old infant) were isolated from faeces of breast-fed infants from Thailand and were shown to have capacity to inhibit *Clostridiodes difficile* and exhibit anti-inflammatory properties [43, 44]. RNA polymerase sigma factor 54 (RpoN) was shown to regulate virulence genes, motility, quorum sensing and also tolerance to antibiotics in *Psudomonas aeruginosa* [45]. Similarly, transcription by sigma 54 holoenzyme was shown to be activated by phosphorylated NtrC oligomers [46]. The role of RNA polymerase sigma-54 factor and NtrC transcriptional regulator in the pathogenicity of the clade of 5 *L. rhamnosus* clinical isolates is yet to be ascertained.

Potential significance could also be attached to the iron ABC transporter permease, iron ABC transporter substrate-binding protein, two component sensor kinase with ferric iron transporter and isochorismatase exclusively conserved between the clade of 5 and *L. rhamnosus* L31. In *Acinetobacter baumannii* a critical correlation has been shown between isochorismatase, siderophore-mediated ferric iron acquisition and autophagy [47]. On the other hand, in *Pseudomonas aeruginosa*, isochorismatase is involved in the biosynthesis of an antimicrobial compound, phenazine, that may offer competitive advantage to this opportunistic pathogen [48].

IS91 family transposase, another unique functional ortholog closely associated with clade of 5, was detected only in JWHC01 (*L. rhamnosus* strain 308 from saliva of a healthy female), AMC010 from stools of a healthy infant and E800 (from faeces). IS91 family transposase, uniquely employs rolling circle transposition in horizontal gene transfer [49, 50]. IS91 insertion sequence has also been identified in Gram negative organisms and a link between IS91 family transposase and pathogenicity and virulence- related genes has been demonstrated for *E. coli* [51, 52]. In the clade of 5, multiple genes designated as encoding hypothetical proteins are located adjacent to the gene encoding IS91 family transposase. Further studies will be required to elucidate the significance of this genic cluster.

## Conclusion

*In-silico* analysis of the genomes of the clade of 5 clinical isolates highlighted a potentially unique complement of genes of clinical relevance. Except for the near identical EPS cluster, with exclusivity defined by five EPS orthologs, no other gene sets were specifically conserved between the clade of 5 and the probiotic strains, *L. rhamnosus* BPL5 and BPL15. Candidates emerging from the distinctive set of 58 genes identified in the clade of 5 include RNA polymerase sigma-54 factor (RpoN), transcriptional regulators, XRE family, NtrC, MutR, iron ABC transporter permease, iron ABC transporter substrate- binding protein and two component sensor kinase with Ferric iron transporter. Others include isochorismatase, DEAD/DEAH box helicase (Type III restriction protein res subunit) associated with remodelling and unwinding of RNA [53], helix-turn helix (HTH) domain containing proteins and PRD domain containing proteins as regulatory domains for PTS carbohydrate metabolism. The BREX defense system, toxin-antitoxin system and IS91 transposase and the surrounding group of phage-related and hypothetical proteins could signal the presence of novel defense systems [54] as components of pathogenicity islands in the clade of 5. Functional co-ordination between different defense systems in addition to the distinctive EPS cluster and associated alpha-L-fucosidase, L-fucose isomerase, metal-independent alpha-mannosidase, HXXEE domain-containing protein and thymidylate synthase, may govern conditional opportunistic pathology associated with *L. rhamnosus*. Further studies could disclose whether the complex interplay between the 58 unique and potentially functional orthologs of the clade of 5 could serve as a model for opportunistic virulence .

## Methods

### *L. rhamnosus* and *Lactobacillus* spp. genome sequences

Genome sequences of *L. rhamnosus* LRHMDP2 and LRHMDP3 isolated from the early stages of dental pulp infection [7, 55], *L. rhamnosus* 699_LRHA and 708_LRHA isolated from bronchoalveolar lavage [6], *L. rhamnosus* BPL5, originating from the vagina of a healthy women [24] and BPL15 and *Lactobacillus* spp. HMSC077C11 isolated from a neck abscess, formed part of this study (Table 4). All other *L. rhamnosus* genomes, which have been sequenced and deposited in NCBI, were used for a comprehensive comparative genomic analysis (Table S1).

**Table 4:** Genome features of *L. rhamnosus* strains in this study

***L. rhamnosus*** / ***Lactobacillus*** spp.	Source	Genome	BioProject / GenBank reference	Genome size (Mbp)	No. of genes	Proteins	Release year	Sequencing technology	Coverage
*Lactobacillus rhamnosus* LRHMDP2	Oral / Dental pulp	contig	PRJNA169251/ AMQW00000000.1	2.91	2967	2824	2012	Roche GS FLX+	17x
*Lactobacillus rhamnosus* LRHMDP3	Oral / Dental pulp	contig	PRJNA169313 / AMQX00000000.1	2.91	2985	2835	2012	Roche GS FLX+	17x
*Lactobacillus rhamnosus* 699_LRHA	ICU / Bronchoalveolar lavage	contig	PRJNA267549 / JUWQ00000000.1	2.95	2977	2791	2015	Illumina (HiSeq-MiSeq)	53x
*Lactobacillus rhamnosus* 708_LRHA	ICU / Bronchoalveolar lavage	contig	PRJNA267549 / JUWG00000000.1	2.96	3001	2813	2015	Illumina (HiSeq-MiSeq)	53x
*Lactobacillus* spp. HMSC077C11^a^	Neck abscess	Contig	PRJNA296298 / LTIR00000000.1	2.89	2897	2730	2016	Illumina	138x
*L. rhamnosus* BPL5	Vagina of healthy women	complete	PRJNA224116 / NZ-LT220504.1	3.02	3042	2854	2016	PacBio (SMRT)	178x
*L. rhamnosus* BPL15	–	Contig	PRJEB4890 / CBZU000000000.1	3.01	3002	2829	2015	–	–

^a^The genome sequence of *Lactobacillus* spp. HMSC077C11 has been lodged in NCBI as an unnamed isolate not characterized using traditional methods and is clearly distinct from currently recognized species

### *L. rhamnosus* phylogenetic tree

We selected the GToTree ​tool to include *Lactobacillus* sp. HMSC077C11 along with the 4 clinical strains (*L. rhamnosus* LRHMDP2, LRHMDP3, 699_LRHA and 708_LRHA) on the *L. rhamnosus* phylogeny tree. *Lactobacillus* sp. HMSC077C11 has been recently re-classified as *L. rhamnosus* HMSC077C11 in the Genome Taxonomy Database (GTDB) by genome-based species taxonomy study [23] and is not represented on the NCBI *L. rhamnosus* phylogeny tree. The phylogenetic tree built by the GToTree tool retains exact grouping as the NCBI *L. rhamnosus* phylogeny tree (https://www.ncbi.nlm.nih.gov/genome/?term=Lactobacillus+rhamnosus).

GToTree is the newly published bioinformatic tool, that can work with any custom hidden Markov Models (HMMs). It has also included 13 newly generated single-copy gene (SCG) set HMMs for different lineages and levels of resolution, built based on searches of ∼12,000 high-quality bacterial and archaeal genomes. GToTree algorithm is more generic than its predecessors and the tool is highly scalable and user friendly. The tool accepts genome sequences and provides an alignment output and phylogenomic tree based on the specified hidden Markov Models (HMM) profiles [56]. From the available 15 single copy gene (SCG)-set HMMs in GToTree, we used the Firmicutes HMM SCG-set with 119 genes. The tree was viewed using the ‘Interactive tree of life’ web page (https://itol.embl.de/upload.cgi). The genbank, fasta and gff format files required for generating a phylogenetic tree and for subsequent comparative genomic analysis were downloaded using the NCBI web link https://www.ncbi.nlm.nih.gov/genome/doc/ftpfaq/#downloadservice (Table S1).

### Exopolysaccharide (EPS) cluster

EPS cluster proteins from *L. rhamnosus* LRHMDP2 and LRHMDP3 were extracted from NCBI. Identical Protein groups (https://www.ncbi.nlm.nih.gov/ipg/) and the tool Proteinortho was used to identify conservation of orthologous genes across the other *L. rhamnosus* strains. Genomic organization of wzb-wzd synteny of the EPS cluster was ascertained using EasyFig, a python application for creating linear comparison figures of multiple genomic loci for displaying the similarities and differences within the ‘wzb-wzd’ synteny EPS cluster locus across the set of selected *L. rhamnosus* strains (LRHMDP2, LRHMDP3, 699_LRHA, 708_LRHA, BPL5 and BPL15 and *Lactobacillus* spp. HMSC077C11 [57]. The EasyFig tool provides a graphical user interface to upload individual Genbank formatted files. Pairwise blastn comparisons were conducted and the blast outputs were used to generate syntenical visualizations of the EPS clusters across selected *L. rhamnosus* strains together with gene orientations.

### Comparative genome-wide analysis

Proteinortho tool was used to identify conservation of orthologous genes across selected *L. rhamnosus* isolates for both whole genome comparisons and for specific analysis of EPS cluster related genes [58]. Proteins specifically conserved in the clade of 5 clinical isolates were further confirmed for their specificity by tblastn alignments (protein sequences) against *L. rhamnosus* genomes, to identify false positives and negatives (due to missing gene annotations in individual genomes) and any other anomalies. Representation of the distinct set of protein orthologs based on the tool ProteinOrtho and tblastn differentiating clade of 5 from *L. rhamnosus* BPL5 and BPL15 was ascertained further in the remaining *L. rhamnosus* genome sequences.

## Supplementary information

**Additional file 1: Table S1.** List of *L. rhamnosus* strains downloaded for phylogenetic tree.

**Additional file 2: Table S2.** Protein orthologs exclusively shared between *L. rhamnosus* LRHMDP2, LRHMDP3, 699_LRHA, 708_LRHA and *Lactobacillus* spp. HMSC077C11 in comparison to *L. rhamnosus* BPL5 and BPL15. Identified using ProteinOrtho and tblastn.

**Additional file 3: Table S3.**

## Data Availability

The genbank, fasta and gff format files required for generating a phylogenetic tree and for subsequent comparative genomics analysis were downloaded using the NCBI web link https://www.ncbi.nlm.nih.gov/genome/doc/ftpfaq/#downloadservice (Table S1). Further data analysis is supported by supplementary files.

## Abbreviations

BREX: Bacteriophage exclusion
Cas: CRISPR associated system
CRISPR: Clustered Regularly Interspaced Short Palindromic Repeat
EPS: Exopolysaccharide
GRAS: Generally recognized as safe
HMM: Hidden Markov Models
ICU: Intensive care unit
SCG: Single copy gene
TA: Toxin-antitoxin
Wzb: Tyrosine protein phosphatase
Wzd: Tyrosine-protein kinase modulator EpsC
