# Supplementary material for: Intra-species variation within Lactobacillus rhamnosus correlates to beneficial or harmful outcomes: lessons from the oral cavity
Source: BMC Genomics. 2020 Sep 24;21:661. doi: 10.1186/s12864-020-07062-3 (PMC7513527; doi:10.1186/s12864-020-07062-3)
Supplement: Supplementary file 1 — Additional file 1: Table S1. List of L. rhamnosus strains downloaded for phylogenetic tree. [file 12864_2020_7062_MOESM1_ESM.docx]

**Table S1. List of *L. rhamnosus* strains downloaded for phylogenetic tree**

|  |  |
| --- | --- |
| **Strain ID** | **GeneBank file name** |
| HMSC077C11 | GCA_001809975.1_ASM180997v1_genomic.gbff.gz |
| Lrh46 | GCA_002103215.1_ASM210321v1_genomic.gbff.gz |
| Lr044 | GCA_001044105.1_ASM104410v1_genomic.gbff.gz |
| 870_LRHA | GCA_001066715.1_ASM106671v1_genomic.gbff.gz |
| ASCC 3029 | GCA_001831235.1_ASM183123v1_genomic.gbff.gz |
| Lrh16 | GCA_001657085.1_ASM165708v1_genomic.gbff.gz |
| Lrh8 | GCA_001656535.1_ASM165653v1_genomic.gbff.gz |
| ATCC 21052 | GCA_000235865.1_ASM23586v1_genomic.gbff.gz |
| AMBR3 | GCA_901830385.1_AMBR3_genomic.gbff.gz |
| Lrh7 | GCA_001656815.1_ASM165681v1_genomic.gbff.gz |
| LR-B1 | GCA_004010975.1_ASM401097v1_genomic.gbff.gz |
| LR2 | GCA_003046115.1_ASM304611v1_genomic.gbff.gz |
| Lrh43 | GCA_001657255.1_ASM165725v1_genomic.gbff.gz |
| Lc 705 | GCA_000026525.1_ASM2652v1_genomic.gbff.gz |
| Lrh23 | GCA_001656635.1_ASM165663v1_genomic.gbff.gz |
| AMBR6 | GCA_901830355.1_AMBR6_genomic.gbff.gz |
| Lrh47 | GCA_002103185.1_ASM210318v1_genomic.gbff.gz |
| INIA P540 | GCA_901971795.1_INIA_P540_genomic.gbff.gz |
| P3 | GCA_002406785.1_ASM240678v1_genomic.gbff.gz |
| Lrh12 | GCA_001657155.1_ASM165715v1_genomic.gbff.gz |
| P5 | GCA_002406715.1_ASM240671v1_genomic.gbff.gz |
| CASL | GCA_000226235.1_ASM22623v1_genomic.gbff.gz |
| 893_LRHA | GCA_001067625.1_ASM106762v1_genomic.gbff.gz |
| DS3_11 | GCA_003052985.1_ASM305298v1_genomic.gbff.gz |
| DS13_11 | GCA_003052945.1_ASM305294v1_genomic.gbff.gz |
| DS9_11 | GCA_003061625.1_ASM306162v1_genomic.gbff.gz |
| R709 | GCA_002027355.1_ASM202735v1_genomic.gbff.gz |
| Lr071 | GCA_001044015.1_ASM104401v1_genomic.gbff.gz |
| LR-B2 | GCA_004125395.1_ASM412539v1_genomic.gbff.gz |
| Lrh33 | GCA_001657195.1_ASM165719v1_genomic.gbff.gz |
| P4 | GCA_002406745.1_ASM240674v1_genomic.gbff.gz |
| Lr053 | GCA_001044085.1_ASM104408v1_genomic.gbff.gz |
| IBL027 | GCA_002238035.1_ASM223803v1_genomic.gbff.gz |
| AMBR7 | GCA_901830365.1_AMBR7_genomic.gbff.gz |
| R0011 | GCA_000235785.2_ASM23578v2_genomic.gbff.gz |
| LRHMDP2 | GCA_000311945.1_ASM31194v1_genomic.gbff.gz |
| Lrh39 | GCA_002103145.1_ASM210314v1_genomic.gbff.gz |
| 24 | GCA_000743075.1_ASM74307v1_genomic.gbff.gz |
| 390_LRHA | GCA_001064785.1_ASM106478v1_genomic.gbff.gz |
| Lrh21 | GCA_001656995.1_ASM165699v1_genomic.gbff.gz |
| Lrh42 | GCA_001657205.1_ASM165720v1_genomic.gbff.gz |
| UMB0004 | GCA_002848015.1_ASM284801v1_genomic.gbff.gz |
| LGR-1 | GCA_900604925.1_LGR1_genomic.gbff.gz |
| 708_LRHA | GCA_001067025.1_ASM106702v1_genomic.gbff.gz |
| Lrh5 | GCA_001656845.1_ASM165684v1_genomic.gbff.gz |
| HCT70 | GCA_001756565.1_ASM175656v1_genomic.gbff.gz |
| 944_LRHA | GCA_001068215.1_ASM106821v1_genomic.gbff.gz |
| co_0103 | GCA_004167055.1_ASM416705v1_genomic.gbff.gz |
| 40f | GCA_001044405.1_ASM104440v1_genomic.gbff.gz |
| LRHMDP3 | GCA_000311965.1_ASM31196v1_genomic.gbff.gz |
| Lrh17 | GCA_001657075.1_ASM165707v1_genomic.gbff.gz |
| CRL1505 | GCA_000414365.1_454ScaffoldContigs1505_genomic.gbff.gz |
| Lrh44 | GCA_001657235.1_ASM165723v1_genomic.gbff.gz |
| ARJD | GCA_003573615.1_ASM357361v1_genomic.gbff.gz |
| Lrh9 | GCA_001656785.1_ASM165678v1_genomic.gbff.gz |
| Lrh15 | GCA_001656715.1_ASM165671v1_genomic.gbff.gz |
| Lrh4 | GCA_001656875.1_ASM165687v1_genomic.gbff.gz |
| LOCK900 | GCA_000418475.1_ASM41847v1_genomic.gbff.gz |
| DSM 20021 | GCA_001435405.1_ASM143540v1_genomic.gbff.gz |
| Lrh22 | GCA_001656655.1_ASM165665v1_genomic.gbff.gz |
| AMBR1 | GCA_901830405.1_AMBR1_genomic.gbff.gz |
| LOCK908 | GCA_000418495.1_ASM41849v1_genomic.gbff.gz |
| Lrh13 | GCA_001657135.1_ASM165713v1_genomic.gbff.gz |
| 51B | GCA_000699985.1_ASM69998v1_genomic.gbff.gz |
| Lr108 | GCA_001044025.1_ASM104402v1_genomic.gbff.gz |
| NCTC13710 | GCA_900636875.1_44927_D01_genomic.gbff.gz |
| GG | GCA_003353455.1_ASM335345v1_genomic.gbff.gz |
| Lrh10 | GCA_001657165.1_ASM165716v1_genomic.gbff.gz |
| BPL5 | GCA_900070175.1_Lactobacillus_rhamnosus_CECT_8800_genomic.gbff.gz |
| Lr138 | GCA_001044075.1_ASM104407v1_genomic.gbff.gz |
| MTCC 5462 | GCA_000195375.2_ASM19537v2_genomic.gbff.gz |
| DS15_11 | GCA_003061645.1_ASM306164v1_genomic.gbff.gz |
| DS18_11 | GCA_003052925.1_ASM305292v1_genomic.gbff.gz |
| R19-3 | GCA_001645615.1_ASM164561v1_genomic.gbff.gz |
| Lrh26 | GCA_001656605.1_ASM165660v1_genomic.gbff.gz |
| 526_LRHA | GCA_001063655.1_ASM106365v1_genomic.gbff.gz |
| LR-S | GCA_004125475.1_ASM412547v1_genomic.gbff.gz |
| L34 | GCA_000784375.1_L34_genomic.gbff.gz |
| LRB | GCA_001721925.1_ASM172192v1_genomic.gbff.gz |
| CLS17 | GCA_000932035.1_ASM93203v1_genomic.gbff.gz |
| FAM 20558 | GCA_005864245.1_ASM586424v1_genomic.gbff.gz |
| DSM 14870 | GCA_002287945.1_ASM228794v1_genomic.gbff.gz |
| Lrh2 | GCA_001657025.1_ASM165702v1_genomic.gbff.gz |
| AMC143 | GCA_001982425.1_ASM198242v1_genomic.gbff.gz |
| DS14_11 | GCA_003061705.1_ASM306170v1_genomic.gbff.gz |
| ATCC 8530 | GCA_000233755.1_ASM23375v1_genomic.gbff.gz |
| Lrh28 | GCA_001656925.1_ASM165692v1_genomic.gbff.gz |
| TMC3115 | GCA_003129615.1_ASM312961v1_genomic.gbff.gz |
| 4928STDY7387919 | GCA_902166035.1_26009_2_28_genomic.gbff.gz |
| DS22_11 | GCA_003061605.1_ASM306160v1_genomic.gbff.gz |
| DS4_11 | GCA_003052965.1_ASM305296v1_genomic.gbff.gz |
| Lrh32 | GCA_001656545.1_ASM165654v1_genomic.gbff.gz |
| HN001 | GCA_000173255.2_ASM17325v2_genomic.gbff.gz |
| NRRL B-442 | GCA_002849515.1_ASM284951v1_genomic.gbff.gz |
| 389_LRHA | GCA_001063295.1_ASM106329v1_genomic.gbff.gz |
| PEL6 | GCA_000712515.1_PEL6_genomic.gbff.gz |
| Lrh1 | GCA_001656755.1_ASM165675v1_genomic.gbff.gz |
| 186_LRHA | GCA_001062885.1_ASM106288v1_genomic.gbff.gz |
| Lrh6 | GCA_001656835.1_ASM165683v1_genomic.gbff.gz |
| LMS2-1 | GCA_000160175.1_ASM16017v1_genomic.gbff.gz |
| 769_LRHA | GCA_001067215.1_ASM106721v1_genomic.gbff.gz |
| Lrh29 | GCA_001656585.1_ASM165658v1_genomic.gbff.gz |
| LR231 | GCA_000508405.1_Lac_rha231_genomic.gbff.gz |
| AMC010 | GCA_001982435.1_ASM198243v1_genomic.gbff.gz |
| AMBR5 | GCA_901830425.1_AMBR5_genomic.gbff.gz |
| 5-Jan | GCA_004122925.1_ASM412292v1_genomic.gbff.gz |
| ASCC 3018 | GCA_001831215.1_ASM183121v1_genomic.gbff.gz |
| ASCC 290 | GCA_001590655.1_ASM159065v1_genomic.gbff.gz |
| L35 | GCA_000784395.1_L35_genomic.gbff.gz |
| L156.4 | GCA_001991035.1_ASM199103v1_genomic.gbff.gz |
| ATCC 11443 | GCA_003433395.1_ASM343339v1_genomic.gbff.gz |
| LR863 | GCA_003129645.1_ASM312964v1_genomic.gbff.gz |
| UBLR-58 | GCA_004798455.1_ASM479845v1_genomic.gbff.gz |
| LR-GG-MoProbi | GCA_004125465.1_ASM412546v1_genomic.gbff.gz |
| Pen | GCA_002076955.1_ASM207695v1_genomic.gbff.gz |
| GR-1 | GCA_002762445.1_ASM276244v1_genomic.gbff.gz |
| Lrh25 | GCA_001656975.1_ASM165697v1_genomic.gbff.gz |
| OGTX02 | GCA_900248175.2_ASM90024817v2_genomic.gbff.gz |
| Lrh19 | GCA_001656685.1_ASM165668v1_genomic.gbff.gz |
| 214_LRHA | GCA_001062955.1_ASM106295v1_genomic.gbff.gz |
| ATCC 53103 | GCA_000011045.1_ASM1104v1_genomic.gbff.gz |
| Lr073 | GCA_001043995.1_ASM104399v1_genomic.gbff.gz |
| LR-CVC | GCA_004125455.1_ASM412545v1_genomic.gbff.gz |
| Lrh27 | GCA_001656945.1_ASM165694v1_genomic.gbff.gz |
| 2166 | GCA_000466865.2_ASM46686v2_genomic.gbff.gz |
| DS12_11 | GCA_003061665.1_ASM306166v1_genomic.gbff.gz |
| B1 | GCA_002406705.1_ASM240670v1_genomic.gbff.gz |
| JWHC01 | GCA_000814485.1_ASM81448v1_genomic.gbff.gz |
| P1 | GCA_002406795.1_ASM240679v1_genomic.gbff.gz |
| 319_LRHA | GCA_001064515.1_ASM106451v1_genomic.gbff.gz |
| SCT-10-10-60 | GCA_002960215.1_ASM296021v1_genomic.gbff.gz |
| 906_LRHA | GCA_001067885.1_ASM106788v1_genomic.gbff.gz |
| 979_LRHA | GCA_001068015.1_ASM106801v1_genomic.gbff.gz |
| ASCC 3016 | GCA_001831225.1_ASM183122v1_genomic.gbff.gz |
| RI-004 | GCA_001981725.1_ASM198172v1_genomic.gbff.gz |
| Lrh24 | GCA_001657005.1_ASM165700v1_genomic.gbff.gz |
| AMBR4 | GCA_901830395.1_AMBR4_genomic.gbff.gz |
| Lrh45 | GCA_001657245.1_ASM165724v1_genomic.gbff.gz |
| Lrh30 | GCA_001656895.1_ASM165689v1_genomic.gbff.gz |
| L31 | GCA_000784405.1_L31_genomic.gbff.gz |
| Lrh34 | GCA_001656765.1_ASM165676v1_genomic.gbff.gz |
| DS17_11 | GCA_003061565.1_ASM306156v1_genomic.gbff.gz |
| BFE5264 | GCA_001988935.1_ASM198893v1_genomic.gbff.gz |
| 541_LRHA | GCA_001065365.1_ASM106536v1_genomic.gbff.gz |
| E800 | GCA_000712495.1_E800_genomic.gbff.gz |
| Lrh11 | GCA_001656735.1_ASM165673v1_genomic.gbff.gz |
| ASCC 1521 | GCA_001831275.1_ASM183127v1_genomic.gbff.gz |
| 1.032 | GCA_006151905.1_ASM615190v1_genomic.gbff.gz |
| 313 | GCA_001044415.1_ASM104441v1_genomic.gbff.gz |
| Lrh18 | GCA_001657055.1_ASM165705v1_genomic.gbff.gz |
| 988_LRHA | GCA_001068045.1_ASM106804v1_genomic.gbff.gz |
| PEL5 | GCA_000712505.1_PEL5_genomic.gbff.gz |
| K32 | GCA_000735255.1_ASM73525v1_genomic.gbff.gz |
| Lrh31 | GCA_001656575.1_ASM165657v1_genomic.gbff.gz |
| GG (ATCC 53103) | GCA_000026505.1_ASM2650v1_genomic.gbff.gz |
| Lr032 | GCA_001044095.1_ASM104409v1_genomic.gbff.gz |
| BPL15 | GCA_001368735.1_CECT8361_genomic.gbff.gz |
| Lr140 | GCA_001044005.1_ASM104400v1_genomic.gbff.gz |
| 699_LRHA | GCA_001066975.1_ASM106697v1_genomic.gbff.gz |
| INIA P344 | GCA_901971785.1_INIA_P344_genomic.gbff.gz |
| Lrh14 | GCA_001657115.1_ASM165711v1_genomic.gbff.gz |
| WQ2 | GCA_002025085.1_ASM202508v1_genomic.gbff.gz |
| 784_LRHA | GCA_001067335.1_ASM106733v1_genomic.gbff.gz |
| 943_LRHA | GCA_001068195.1_ASM106819v1_genomic.gbff.gz |
| LR5 | GCA_002286235.1_ASM228623v1_genomic.gbff.gz |
| 4B15 | GCA_002158925.1_ASM215892v1_genomic.gbff.gz |
| JCM 1136 | GCA_000615245.1_ASM61524v1_genomic.gbff.gz |
| Lrh20 | GCA_001656675.1_ASM165667v1_genomic.gbff.gz |
| Lrh3 | GCA_001656915.1_ASM165691v1_genomic.gbff.gz |
| CNCM-I-3698 | GCA_001005625.1_ASM100562v1_genomic.gbff.gz |
| Lrh38 | GCA_002103155.1_ASM210315v1_genomic.gbff.gz |
| NCTC13764 | GCA_900636965.1_45296_G02_genomic.gbff.gz |
| 116 | GCA_000801045.1_ASM80104v1_genomic.gbff.gz |
|  | [https://www.ncbi.nlm.nih.gov/genome/doc/ftpfaq/#downloadservice](https://www.ncbi.nlm.nih.gov/genome/doc/ftpfaq/%23downloadservice) |
