# Supplementary material for: Intra-species variation within Lactobacillus rhamnosus correlates to beneficial or harmful outcomes: lessons from the oral cavity
Source: BMC Genomics. 2020 Sep 24;21:661. doi: 10.1186/s12864-020-07062-3 (PMC7513527; doi:10.1186/s12864-020-07062-3)
Supplement: Supplementary file 2 — Additional file 2: Table S2. Protein orthologs exclusively shared between L. rhamnosus LRHMDP2, LRHMDP3, 699_LRHA, 708_LRHA and Lactobacillus spp. HMSC077C11 in comparison to L. rhamnosus BPL5 and BPL15. Identified using ProteinOrtho and tblastn. [file 12864_2020_7062_MOESM2_ESM.docx]

**Table S2.** Protein orthologs exclusively shared between *L. rhamnosus* LRHMDP2, LRHMDP3, 699_LRHA, 708_LRHA and *Lactobacillus* spp. HMSC077C11 in comparison to *L. rhamnosus* BPL5 and BPL15. Identified using ProteinOrtho and tblastn

| **Protein IDs** | **Gene description** |
| --- | --- |
| WP_005717709.1 | 4-hydroxy-2-oxoglutarate aldolase / 2-dehydro-3-deoxyphosphogluconate aldolase |
| WP_005715415.1 | ABC-2 transporter permease |
| WP_005715999.1 | AbrB/MazE/SpoVT family DNA-binding domain-containing protein |
| WP_005717725.1 | alpha-L-fucosidase |
| WP_005715325.1 | alpha/beta hydrolase |
| WP_005714953.1 | AraC family transcriptional regulator |
| WP_005717598.1 | ATP-binding protein |
| WP_076638842.1 | bacteriocin |
| WP_005714949.1 | beta-galactosidase subunit alpha |
| WP_005686196.1 | BREX system Lon protease-like protein BrxL |
| WP_005715305.1 | BREX system P-loop protein BrxC |
| WP_005686195.1 | BREX-1 system phosphatase PglZ type A |
| WP_005715915.1 | carbohydrate PTS IIA component |
| WP_005715299.1 | cytosine permease |
| WP_049168901.1 | DNA helicase |
| WP_005684780.1 | DUF1788 domain-containing protein |
| WP_005684779.1 | DUF1819 domain-containing protein |
| WP_005716418.1 | DUF2568 domain-containing protein |
| WP_070586510.1 | DUF262 domain-containing protein sp. HMSC077C11 |
| WP_005714931.1 | DUF2620 domain-containing protein |
| WP_005685750.1 | DUF2992 domain-containing protein |
| WP_080600050.1 | DUF4260 domain-containing protein |
| WP_005715298.1 | DUF917 domain-containing protein |
| WP_005717713.1 | galactonate dehydratase |
| WP_005717726.1 | GntR family transcriptional regulator |
| WP_005717505.1 | helix-turn-helix domain-containing protein |
| WP_032954331.1 | helix-turn-helix domain-containing protein |
| WP_005716405.1 | HXXEE domain-containing protein |
| WP_005715297.1 | hydantoinase/oxoprolinase family protein |
| WP_005715904.1 | ImmA/IrrE family metallo-endopeptidase |
| WP_005716278.1 | iron ABC transporter permease |
| WP_005716273.1 | iron ABC transporter substrate-binding protein |
| WP_005717838.1 | IS91 family transposase |
| WP_005715911.1 | isochorismatase |
| WP_005717692.1 | L-fucose isomerase |
| WP_005714932.1 | membrane protein |
| WP_070586464.1 | MerR family transcriptional regulator sp. HMSC077C11 |
| WP_005717706.1 | metal-independent alpha-mannosidase |
| WP_005715414.1 | N-acetyltransferase |
| WP_005717601.1 | nucleoside-diphosphate sugar epimerase |
| WP_005716841.1 | phage portal protein |
| WP_070586459.1 | phosphotriesterase-related protein sp. HMSC077C11 |
| WP_005714928.1 | PRD domain-containing protein |
| WP_005714930.1 | PRD domain-containing protein |
| WP_032954335.1 | PRD domain-containing protein |
| WP_015764910.1 | pyridoxamine 5-phosphate oxidase family protein |
| WP_005714927.1 | RNA polymerase sigma-54 factor |
| WP_005684771.1 | SDR family NAD(P)-dependent oxidoreductase |
| WP_070586541.1 | SEC10/PgrA surface exclusion domain-containing protein sp. HMSC077C11 |
| WP_080600030.1 | site-specific integrase |
| WP_032954600.1 | thymidylate synthase |
| WP_005714910.1 | transaldolase |
| WP_005714895.1 | transketolase |
| WP_005716270.1 | Two-component sensor kinase associated with ferric iron transporter |
| WP_005717600.1 | type 1 glutamine amidotransferase domain-containing protein |
| WP_005716818.1 | type III restriction protein res subunit |
| WP_005715906.1 | XRE family transcriptional regulator |
| WP_005714936.1 | YhfX family PLP-dependent enzyme |
| WP_070586538.1 | hypothetical protein sp. HMSC077C11 |
| WP_005716134.1 | hypothetical protein |
| WP_005686759.1 | hypothetical protein |
| WP_005711376.1 | hypothetical protein |
| WP_005711378.1 | hypothetical protein |
| WP_005714822.1 | hypothetical protein |
| WP_005714881.1 | hypothetical protein |
| WP_005714929.1 | hypothetical protein |
| WP_005714935.1 | hypothetical protein |
| WP_005714954.1 | hypothetical protein |
| WP_005714987.1 | hypothetical protein |
| WP_005715020.1 | hypothetical protein |
| WP_005715626.1 | hypothetical protein |
| WP_005715794.1 | hypothetical protein |
| WP_005715807.1 | hypothetical protein |
| WP_005715909.1 | hypothetical protein |
| WP_005716036.1 | hypothetical protein |
| WP_005716040.1 | hypothetical protein |
| WP_005716343.1 | hypothetical protein |
| WP_005716521.1 | hypothetical protein |
| WP_005716522.1 | hypothetical protein |
| WP_005716741.1 | hypothetical protein |
| WP_005716827.1 | hypothetical protein |
| WP_005716829.1 | hypothetical protein |
| WP_005716831.1 | hypothetical protein |
| WP_005716834.1 | hypothetical protein |
| WP_005716836.1 | hypothetical protein |
| WP_005716847.1 | hypothetical protein |
| WP_005716850.1 | hypothetical protein |
| WP_005717526.1 | hypothetical protein |
| WP_005717614.1 | hypothetical protein |
| WP_005717666.1 | hypothetical protein |
| WP_005717715.1 | hypothetical protein |
| WP_005717731.1 | hypothetical protein |
| WP_005717840.1 | hypothetical protein |
| WP_014568922.1 | hypothetical protein |
| WP_014569053.1 | hypothetical protein |
| WP_032954323.1 | hypothetical protein |
| WP_032954422.1 | hypothetical protein |
| WP_032954440.1 | hypothetical protein |
| WP_032954532.1 | hypothetical protein |
| WP_032954616.1 | hypothetical protein |
| WP_032955502.1 | hypothetical protein |
| WP_049168580.1 | hypothetical protein |
| WP_049168767.1 | hypothetical protein |
| WP_080599966.1 | hypothetical protein |
| WP_080600006.1 | hypothetical protein |
| WP_080600007.1 | hypothetical protein |
| WP_080600046.1 | hypothetical protein |
